# Prevalence of mistreatment in veterinary medical education; a survey of 3rd year veterinary students at a single university

**DOI:** 10.1186/s12909-024-06610-9

**Published:** 2025-02-25

**Authors:** Katherine H. Barnes, Stephanie Johnson, Chin-Chi Liu, Karanvir S. Aulakh

**Affiliations:** 1 Department of Small Animal Clinical Sciences, Texas A&M University, Raymond Stotzer Parkway, College Station, 77845 College Station, TX USA; 2 Veterinary Clinical Sciences, Louisiana State University, Skip Bertman Drive, 70803 Baton Rouge, USA; 3 Veterinary Specialty Hospital by Ethos Veterinary Health, BVSc & AH, 1901 Douglas Blvd, 95678 Roseville, USA

**Keywords:** Quality of life, Mistreatment, Veterinary education

## Abstract

**Background:**

Improving quality of life in veterinary medicine has emerged as an important topic. One factor which may influence quality of life in medical education is mistreatment (verbal abuse, power abuse, and physical abuse to establish and maintain a power hierarchy). Although it has been documented in medical education, its occurrence in veterinary education is not yet known.

**Methods:**

Third year veterinary students were invited to participate in an anonymous and voluntary survey on mistreatment during the preclinical and clinical education. Students were asked if they witnessed and/or experienced a mistreatment, the type of mistreatment, and the person responsible for administering the mistreatment. Quality of life scores were obtained using the Medical Outcomes Trust short form questionnaire (SF-36). An analysis of variance (ANOVA) model followed by pairwise Least Significant Difference post hoc comparison was used to investigate the relationship variables.

**Results:**

Fifty-five of 60 students (91.7%) that completed the questions on mistreatment indicated that they experienced and/or witnessed a mistreatment during veterinary education. 92% of students that experienced a mistreatment said that it interfered with the learning environment but only 29% of students reported the mistreatment at the time that it occurred. The most common mistreatment was public humiliation (78.3%) followed by special treatment based on gender (63.3%) and racially or ethnically offensive remarks (28.3%). Only racially or ethnically insensitive remarks were associated with a lower quality of life score in the social functioning category (*p* = 0.0131).

**Conclusions:**

Mistreatment frequently occurred during the preclinical and clinical education of veterinary students in this population and interfered with the learning environment. The only mistreatment associated with a lower quality of life score was racially insensitive remarks. Programs to educate students and clinicians/staff about mistreatment and how to handle education in a stressful clinical and a preclinical setting may be of benefit in the future.

**Clinical trial number:**

Not applicable.

**Supplementary Information:**

The online version contains supplementary material available at 10.1186/s12909-024-06610-9.

## Background

Veterinary professionals are often faced with high levels of stress during their career. In 2013, 85% of veterinarians surveyed at the American Veterinary Medical Association conference noted that stress and burnout were among the most important issues affecting the veterinary community [1, 2]. Documented sequelae to high stress and burnout in the medical field include fatigue, appetite changes, cold/flu-like symptoms, irritability, low career satisfaction, increased medical errors, and suboptimal patient care [1–4]. Additionally, a 2019 article showed that the mortality rate for veterinarians due to suicide was proportionately higher than the general population and female veterinarians were 3.4-5 times more likely to die by suicide compared to the general population further emphasizing the importance of this issue [5].

High stress and burnout in veterinary medicine exist not only in the workplace but also prior to graduation from veterinary school [6, 7]. Factors contributing to stress for veterinary students are numerous and include sleep deprivation, excessive workload, inadequate support, and the desire to excel on academic evaluations [1, 7, 8]. For medical students, another factor documented to increase stress and burnout is mistreatment [9–14]. Mistreatment is an umbrella term which reflects many actions intended to intimidate and establish power over students or young doctors (such as residents or interns) including verbal, power, or physical abuse and public humiliation/embarrassment [9, 11, 15–17]. In addition to elevating stress, it has been linked to a decreased confidence in clinical abilities [16]. Although high rates of mistreatment have been documented in medical education (38–93%), very few of these incidences were reported at the time that they occurred [18, 19]. If mistreatment is documented, interventional programs can be implemented to aid is reporting and minimize the occurrence and detrimental effects of this practice [12, 13, 20, 21].

The prevalence of mistreatment in veterinary education, and its effects on students’ well-being, is largely unknown. Therefore, the aim of this study was to evaluate student perceived mistreatment during veterinary education at a university and determine the impact, if any, on quality of life. We hypothesized that student perceived mistreatment would occur with high prevalence in this population and that experiencing mistreatment would be associated with a lower quality of life.

## Methods

This study was reviewed and approved by the Institutional Review Board at Louisiana State University. Veterinary students in the class of 2018 and 2019 at the same university were invited to participate in the study. There was no pilot study prior to data collection. Invitations included a cover letter sent via email which contained a link to an online survey (administered by Survey Monkey^1^) and indicated that all responses would be kept confidential and anonymous. Email reminders to complete the survey were sent out weekly for a total of 4 weeks. The class of 2018 was invited to take the survey in December 2017 and the class of 2019 was invited to take the survey in December, 2018. Students at this particular university start clinical rotations in January of the 3rd year so participants had the opportunity to be on clinics for approximately 1 year prior to completion of the survey. There was no incentive provided to students to who completed the study. Informed consent was obtained from all participants prior to starting the survey. This study adhered to the declaration of Helsinki. (Clinical trial number: not applicable).

### Surveys

Mistreatment was assessed using a questionnaire modified from a previous study (supplemental material) [20]. The first question in the survey defined mistreatment by the following statement: Mistreatment in medical education has been described by intimidation, undermining, unjustified criticism, attempts to publicly humiliate or belittle, making inappropriate jokes, eye rolling, physical abuse (slapping, kicking, or hitting), discrimination based on gender, ethnicity, or sexuality in the learning environment. Students were then asked if they experienced mistreatment or if they witnessed a mistreatment to a third party. Mistreatments were further divided into different categories and the students were asked to select the type of mistreatment they experienced and/or witnessed: verbal abuse, physical abuse, sexual harassment, ethnic mistreatment, abuse of power (such as being threatened about a recommendation, grade, or career), or other (free text box). Additionally, students were asked to identify the role of the person who delivered the mistreatment (such as a clinical faculty, preclinical faculty, intern, resident, veterinary technician, staff, client, or other student) and whether or not the incident was reported. If the mistreatment was not reported, the student was prompted to give the reason for not reporting the mistreatment by selecting one of the following: the incident did not seem important enough to report, I resolved the issue myself, I did not think anything could be done about it, fear of reprisal, I did not know where to report it, I reported all incidents, or other (free text box). The frequency of the mistreatment was also recorded: 0, 1–2 mistreatments, 3–5 mistreatments, 5–10 mistreatments, or greater than 10 mistreatments.

Quality of life was assessed via the Medical Outcomes Trust short form questionnaire (SF-36) previously used across multiple populations [22–25]. The questionnaire assessed 8 different realms of overall physical and mental health including physical functioning, role limitations (physical), role limitation (emotional), bodily pain, social functioning, emotional well-being, energy-fatigue, and general health [23]. Surveys were scored according to published instructions to provide an overall score (0-100) for each of the 8 Sect. [26]. Higher scores indicated a more favorable response (for instance, a higher score in the bodily pain category would indicate lower pain).

Questions about demographic information were included at the end of the survey (age and gender). Students were also asked to identify their plan upon graduation by choosing one of the following: internship with plans for specialization (residency), internship followed by private practice, private practice, research or advanced degree without any clinical work, or other (free text box). Additionally, students were asked how confident they felt in the ability to succeed in clinical veterinary medicine after graduation: extremely confident, moderately confident, unsure, not very confident, or not at all confident.

### Statistical analysis

Data analyses were performed with commercially available statistical software^2^. Primary outcomes assessed included the 8 quality of life scores, mistreatment in veterinary education, plan after graduation, and confidence level. Following collection of data, an analysis of variance (ANOVA) model followed by pairwise Least Significant Difference post hoc comparison was used to investigate the relationship between continuous variables (quality of life scores) and other ordinal or categorical variables (including mistreatment, age, gender, confidence, and plan after graduation). The residuals from all ANOVA models were checked and confirmed for normality with the Shapiro- Wilk test. Assumptions of these models (linearity, normality of residuals, and homoscedasticity of residuals) and influential data points were also assessed by examining standardized residual and quantile plots. Chi-Square test of independence was used to investigate the relationships between categorical and ordinal variables. The significance was set at *p* < 0.05.

## Results

### Demographics

One hundred and seventy-seven students were invited to participate in the survey; 88 in the class of 2018 and 89 were in the class of 2019. A total of 56/177 (31.6%) students completed the entire survey. Four additional students completed only the mistreatment portion without answering quality of life questions. One student completed the quality of life questions but not the mistreatment section. This led to a total of 61 surveys analyzed and a response rate of 61/177 (34.5%). Twenty-nine students indicated they were in the class of 2018, 30 students indicated they were in the class of 2019, and 2 students did not answer this question. Out of the 58 students who completed demographic information on gender, 49/58 (84.5%) were female and 9/58 (15.5%) were male. Fifty seven students indicated their age: 22/57 (38.6%) were under 25 years of age, 26/57 (45.6%) were between the age of 26 and 30 years, 6/57 (8.8%) were between the age of 31 and 35 years, 2/57 (3.5%) were between 36 and 40 years of age, and 1/57 (1.8%) was > 41 years of age. Fifty-eight students responded to the question on confidence in clinical abilities: 8/58 (13.8%) were extremely confident, 28/58 (43.1%) were moderately confident, 8/58 (13.8%) were not very confident, and 14/58 (24.1%) were unsure how confident they were. Fifty-eight students indicated their plan for after graduation: internship followed by private practice (4/58, 6.9%), internship with the desire to specialize (residency) (12/58, 20.7%), other (5/58, 8.6%), or private practice (37/58, 63.8%).

### Mistreatment survey results

Fifty-five out of 60 students (91.7%) reported experiencing and/or witnessing a mistreatment during their veterinary education. Eight out of sixty students (13.3%) experienced at least 1 mistreatment, 6/60 students (10%) witnessed at least 1 mistreatment, and 41/60 students (68.3%) both experienced and witnessed mistreatment. Mistreatments occurred 1–2 times (19/60, 31.7%), 3–5 times (19/60, 31.7%), 5–10 times (8/60, 13.3%), and greater than 10 times (8/60, 13.3%). Out of the 55 students that experienced or witnessed a mistreatment, 51 (92.3%) said that it interfered with the learning environment. Mistreatment occurred during preclinical education (2/55, 3.6%), clinical education (28/55, 50.9%), and both preclinical and clinical education (25/55, 45.5%). Sixteen out of fifty-five students (29%) reported at least 1 mistreatment at the time it occurred. Reasons for not reporting the mistreatment included “I did not think anything could be done about it” (34/55 61.8%), “the incident did not seem important enough to report” (15/55, 27.3%), “fear of reprisal” (19/55, 34.5%), “I did not know where to report it” (9/55, 16.4%), and “I resolved the issue myself” (6/55, 10.9%).

Table 1 is a breakdown of mistreatment by type. Clinical faculty, interns, residents, fellows, and veterinary technicians were most frequently reported as being responsible for the mistreatment. However, all categories of personnel (including other students, administrators, and preclinical faculty) along with clients were reported to have mistreated students during veterinary education (Table 2). When students indicated that they experienced or witnessed being subjected to racially or ethnically offensive remarks, other students were most commonly implicated as responsible for the mistreatment.

**Table 1:** Summary of mistreatment type and the number of students who experienced and witnessed, experienced, and witnessed each mistreatment. Measured data represent the number of students reporting each mistreatment along with (%)

Mistreatment type	Experienced and Witnessed	Experienced	Witnessed
Any mistreatment	41 (68%)	8 (13%)	6 (10%)
Public humiliation or Ridicule	30 (50%)	5 (8%)	12 (20%)
Threatened with physical harm	1 (1.7%)	-	1 (1.7%)
Physically harmed	1 (1.7%)	1 (1.7%)	2 (3.3%)
Required to perform personal services	2 (3.3%)	-	4 (6.7%)
Denied opportunities because of gender	8 (13%)	3 (5%)	3 (5%)
Saw other students being given special treatment due to gender	16 (26.7%)	9 (15%)	13 (21.2%)
Denied opportunities because of sexual orientation	1 (1.7%)	1 (1.7%)	1 (1.7%)
Denied opportunities due to ethnicity or race	-	-	3 (5%)
Subjected to unwanted sexual advances	2 (3.3%)	-	5 (8.3%)
Subjected to racially or ethnically offensive remarks	2 (3.3%)	2 (3.3%)	13 (21.2%)
Subjected to offensive remarks due to sexual orientation	1 (1.7%)	1 (1.7%)	8 (13.3%)
Received lower grades based on gender	3 (5%)	1 (1.7%)	-
Received lower grades based on ethnicity or race	-	1 (1.7%)	1 (1.7%)
Received lower grades based on sexual orientation	-	1 (1.7%)	-

**Table 2:** Mistreatment by Personnel. Measured data represent the number of students reporting each mistreatment along with (%)

	Personnel						
Mistreatment type	Preclinical Faculty	Clinical Faculty	Resident, Intern, Fellow	Veterinary Technician	Other Student	Administrator	Client
Public humiliation or Ridicule	15 (25%)	26 (43.3%)	31 (51.7%)	17 (28.3%)	11 (18.3%)	-	10 (16.7%)
Threatened with physical harm	-	1 (1.7%)	-	1 (1.7%)		-	1 (1.7%)
Physically harmed	1 (1.7%)	-	1 (1.7%)	-	1 (1.7%)	-	-
Required to perform personal services	-	6 (10%)	2 (3.3%)	1 (1.7%)	-	-	-
Denied opportunities because of gender	5 (8.3%)	6 (10%)	6 (10%)	5 (8.3%)	2 (3.3%)	1 (1.7%)	-
Saw other students being given special treatment due to gender	16 (26.7%)	19 (31.7%)	22 (36.7%)	15 (25%)	5 (8.3%)	3 (5%)	4 (6.7%)
Denied opportunities because of sexual orientation	-	1 (1.7%)	2 (3.3%)	1 (1.7%)	2 (3.3%)		
Denied opportunities due to ethnicity or race	1 (1.7%)	2 (3.3%)	2 (3.3%)	2 (3.3%)	2 (3.3%)	1 (1.7%)	1 (1.7%)
Subjected to unwanted sexual advances	4 (6.7%)	5 (8.3%)	6 (10%)	-	2 (3.3%)	-	
Subjected to racially or ethnically offensive remarks	3 (5%)	4 (6.7%)	2 (3.3%)	1 (1.7%)	11 (18.3%)	-	1 (1.7%)
Subjected to offensive remarks due to sexual orientation	-	2 (3.3%)	2 (3.3%)	-	8 (13.3%)	-	-
Received lower grades based on gender	-	3 (5%)	1 (1.7%)	1 (1.7%)	1 (1.7%)	-	-
Received lower grades based on ethnicity or race	-	1 (1.7%)	1 (1.7%)	-	-	-	-
Received lower grades based on sexual orientation	-	-	-	1 (1.7%)	-	-	-

### Quality of life survey results

Fifty-seven students completed the quality of life questionnaire. Table 3 lists the mean ± SD for each category. For all categories, higher scores indicate a more favorable response. Significant associations between quality of life scores and other variables are summarized in Table 4. Mistreatments that were reported by 10 or more students were evaluated separately for their effects on quality of life scores. Racially offensive remarks were the only mistreatment shown to have an effect on a quality of life score. Students who experienced and witnessed, or witnessed racially offensive remarks had lower scores for social functioning (12.5 ± 17.5 and 44.2 ± 6.9 respectively) compared to those that did not witness or experience this mistreatment (60.6 ± 3.9, *p* = 0.0098 and *p* = 0.0432 respectively).

**Table 3:** Summary of the mean (± SD) for each of category of the Medical Outcomes Trust short form (SF-36) for the group of students in this study and that of a sample of the general population

SF-36 Category	Students (mean ± SD)
Physical functioning	91.8 ± 10.5
Role limitation - physical	76.8 ± 34.3
Role limitation - emotional	38.9 ± 37.7
Energy-fatigue	29.8 ± 16.8
Emotional well-being	52.3 ± 18.2
Social functioning	53.7 ± 27.0
Bodily Pain	76.1 ± 19.6
General health	62.1 ± 21.0

**Table 4:** Summary of significant relationships between quality of life scores and mistreatment. Quality of life scores are on a range of 0-100 with higher scores indicating a more favorable response (for example, higher score for bodily pain indicates less bodily pain)

Mistreatment	Social Functioning
E/W Racially Insensitive Remarks	12.5 ± 17.5 *
W Racially Insensitive Remarks	(44.2 ± 6.9)*
Did Not E/W Racially insensitive remarks	60.6 ± 3.9†

Scores in the same table column with the same symbol are not significantly different from each other

E/W – Experienced and witnessed

W - Witnessed

## Discussion

To our knowledge, this is the first report of mistreatment perceived by students within veterinary preclinical and clinical education. Based on our results, we accepted our hypothesis that there would be a high prevalence of mistreatment perceived by students in this population and additionally found that one type of mistreatment, experiencing/witnessing racially insensitive remarks, was associated with a lower quality of life score in the social functioning category. Other types of mistreatment were not significantly associated with quality of life scores but occurred throughout the preclinical and clinical years.

Mistreatment in medical education (including verbal abuse, power abuse, sexual harassment, and physical mistreatment) has been reported by 38–93% of students and residents [9–11, 27]. Similarly, we found that 91.7% of students that responded to our survey reported experiencing and/or witnessing a mistreatment with the most common type being public humiliation. Proposed reasons for mistreatment in this learning environment include the structured hierarchy of the hospital or university, failure to report mistreatment when it occurs, fear of reprisal if one does come forward, and acceptance that mistreatment is just part of the educational culture in medicine [18, 19, 28]. Failure to report mistreatment was also documented in the current study with only 29% of students reporting mistreatment at the time when it occurred. The 2 most common reasons for not reporting mistreatment in the current study were “I did not think anything could be done about it” and “fear of reprisal”. Based on these findings, it becomes clear that there must be a method in place to empower students who feel mistreated to speak up and find a solution without fear of negative repercussions.

Second to public humiliation, we found that mistreatments surrounding race and gender were the next most commonly reported. One possible explanation for gender as a central role for mistreatment is the large discrepancy between male and female students enrolled in veterinary school. This gender imbalance may lead to different treatment based on gender, or the perception that others are being treated differently due to gender. Gender based discrimination has been previously documented in medical education and may lead to the perception of unequal educational opportunities between genders [29]. Reasons for the higher level of mistreatment based on racially insensitive remarks is unclear. This type of mistreatment is especially important in this study as it was the only one associated with a lower quality of life score. Students that witnessed and/or experienced racially insensitive remarks had lower scores for social functioning compared to students that did not experience or witness this mistreatment. Given that other students were most commonly implicated in this mistreatment, it is possible that racially insensitive remarks may make someone feel alienated from the rest of the class and less willing to participate in social activities. A previous study from the University of California Las Angeles (UCLA) also reported higher levels of mistreatment related to ethnicity and noted that this type of mistreatment was less likely to be reported compared to mistreatment related to power, sexual harassment, and physical mistreatment [15].

Once mistreatment is documented, all personnel must be on board to initiate change: this includes students, faculty, staff, interns/residents, and administrators. Potential roadblocks to change may be present, especially if clinicians believe that public humiliation and/or intimidation are educational tools [27, 30]. It is possible that educators may rationalize the use of intimidation during education if they believe a student’s behavior will lead to poor patient care, or previous attempts to change the behavior through other means has failed [27]. Although veterinary educators were not surveyed for their perspective on mistreatment, a future study interviewing faculty, interns, residents, and technicians may help to further understand these interactions.

In addition to making clinicians aware of this issue, educating students about learning in a clinical environment is also important. One such training program was trialed at the Stanford University School of Medicine [20]. During this program, 3rd year medical students were educated on the definition of a mistreatment, and also had confidential group discussions on different clinical scenarios. These scenarios included obvious mistreatment (such as public humiliation), but also included high stress scenarios which could be easily misinterpreted (such as a tense situation when dealing with a bleeding patient). Students were encouraged to come forward if they felt uncomfortable and given direct access to personnel who could help. After implementing the program, a lower number of mistreatments were reported and students thought the learning environment and culture had improved. It is possible that a similar program could be of benefit in veterinary medicine and would allow students to understand this novel learning environment and feel comfortable coming forward without fear of reprisal. Improving the learning environment as a whole may also help to improve the culture of the future workplace if students are more aware of how to interact productively in a stressful environment. Although not a guarantee, it seems reasonable to assume that students educated in an environment with healthy interpersonal interactions would carry this atmosphere into the workplace after graduation [31].

The ultimate goal of improving the educational and working environment in veterinary medicine is to improve quality of life for veterinary professionals long term. The quality of life scores for this group of students was particularly low in the emotional categories (role limitation- emotional, emotional well-being) as well as social functioning and energy-fatigue. Possible reasons for this in the clinical environment include managing patients in emotionally stressful situations (such as euthanasia) and interacting with frustrated/emotional clients [32]. In the pre-clinical setting, stress can occur from excessive workload, lack of free time, lack of sleep, lack of exercise, neuroticism, inadequate support, and social isolation [1, 6, 7]. The consequences of high stress levels are multiple and include health related problems, psychological symptoms, and poor job performance [1, 8, 33].

Limitations of this study include the small sample size and a sample limited to one institution. A multi-institutional study in the future would help to document the occurrence of mistreatment in other universities. Another limitation is that the survey was only administered during one part of the year. A previous study showed different emotional exhaustion and burnout rates depending on the year and semester [6]. It is possible that our findings would also change if the survey was administered at a different time point. The students in this study were also asked to report all mistreatment from the previous 3 years of education. Relying on students’ memory over this length of time may have led to an under-reporting of mistreatment. Additionally, not every student responded to the survey. It is possible that students who felt mistreated during their education were more likely to respond to this survey. Obtaining responses from more (or all students) in a class would provide a more accurate assessment of mistreatment in veterinary education. Lastly, clinicians and other educators (such as veterinary technicians) were not surveyed for their impression on mistreatment and quality of life. It is possible that these personnel may have a different impression of mistreatment in the preclinical or clinical setting, or may experience mistreatment of their own from other members of the educational community. Future studies evaluating this topic from their perspective would help investigate the culture of veterinary education in place today.

## Conclusions

This study found that mistreatment frequently occurs during the preclinical and clinical education of veterinary students in this study and interferes with the learning environment. Quality of life scores for veterinary students were low particularly surrounding the categories dealing with emotions and fatigue. The only mistreatment associated with a lower quality of life score was racially insensitive remarks. Programs to educate students and clinicians/staff about mistreatment and how to handle education in a stressful setting may be of benefit in the future.

## Electronic supplementary material

Below is the link to the electronic supplementary material.

Supplementary Material 1

## Data Availability

The datasets used and/or analysed during the current study are available from the corresponding author on reasonable request.

## Footnotes

1 SurveyMonkey, San Mateo, CA.

2 JMP Pro 13, SAS Institute Inc., Cary, NC.

## Abbreviations

ANOVA: Analysis of variance
SD: Standard deviation
