# Supplementary material for: Prevalence of mistreatment in veterinary medical education; a survey of 3rd year veterinary students at a single university
Source: BMC Med Educ. 2025 Feb 25;25:305. doi: 10.1186/s12909-024-06610-9 (PMC11863828; doi:10.1186/s12909-024-06610-9)
Supplement: Supplementary file 1 — Supplementary Material 1 [file 12909_2024_6610_MOESM1_ESM.pdf]

Mistreatment in Veterinary Education 2

Evaluation of mistreatment

* 1. Mistreatment in medical education has been described by intimidation, undermining, unjustified criticism, attempts to publicly humiliate or belittle, making inappropriate jokes, eye rolling, physical abuse (slapping, kicking, or hitting), discrimination based on gender, ethnicity, or sexuality in the learning environment. Please indicate which of the following is most applicable to your experience.

- ○ I have experienced a mistreatment during my preclinical or clinical veterinary education
- ○ I have witnessed another student being subjected to a mistreatment during my preclinical or clinical veterinary education
- ○ I have both experienced and witnessed mistreatments during my preclinical or clinical veterinary education
- ○ I have not experienced or witnessed mistreatments during my preclinical or clinical veterinary education

* 2. Which of the following have you experienced or witnessed during your clinical or preclinical veterinary education. If applicable, please indicate the person responsible for the mistreatment. Select all that apply. If you did not experience or witness a particular mistreatment, please select "N/A".

	I experienced this mistreatment	I witnessed another student subjected to this mistreatment	Preclinical faculty	Clinical faculty	Intern, resident, fellow	Nurse	Student	Administrator	Client	N/A
Public humiliation or ridicule	☐	☐	☐	☐	☐	☐	☐	☐	☐	☐
Threatened with Physical harm	☐	☐	☐	☐	☐	☐	☐	☐	☐	☐
Physically harmed (e.g., slapped, kicked)	☐	☐	☐	☐	☐	☐	☐	☐	☐	☐
Required to perform personal services (e.g., babysitting, shopping)	☐	☐	☐	☐	☐	☐	☐	☐	☐	☐
Denied opportunities for training or rewards solely because of gender	☐	☐	☐	☐	☐	☐	☐	☐	☐	☐

	I experienced this mistreatment	I witnessed another student subjected to this mistreatment	Preclinical faculty	Clinical faculty	Intern, resident, fellow	Nurse	Student	Administrator	Client	N/A
Saw other students being given special treatment because of their gender	☐	☐	☐	☐	☐	☐	☐	☐	☐	☐
Denied opportunities for training or rewards solely because of sexual orientation	☐	☐	☐	☐	☐	☐	☐	☐	☐	☐
Denied opportunities for training or rewards solely because of ethnicity or race	☐	☐	☐	☐	☐	☐	☐	☐	☐	☐
Subjected to unwanted sexual advances	☐	☐	☐	☐	☐	☐	☐	☐	☐	☐
Subjected to racially or ethnically offensive remarks	☐	☐	☐	☐	☐	☐	☐	☐	☐	☐
Subjected to offensive remarks because of sexual orientation	☐	☐	☐	☐	☐	☐	☐	☐	☐	☐
Received lower grades solely based on gender	☐	☐	☐	☐	☐	☐	☐	☐	☐	☐
Received lower grades solely based on ethnicity or race	☐	☐	☐	☐	☐	☐	☐	☐	☐	☐
Received lower grades solely based on sexual orientation	☐	☐	☐	☐	☐	☐	☐	☐	☐	☐

Other (please specify)

[ ]

* 3. How often did any of the above mistreatments occur?

- ◯ Never – I never witnessed or experienced a mistreatment
- ◯ 1-2 times
- ◯ 3-5 times
- ◯ 5-10 times
- ◯ Greater than 10 times

* 4. If you experienced or witnessed a mistreatment, did it occur in the preclinical education or the clinical education?

- ◯ Preclinical
- ◯ Clinical
- ◯ Both
- ◯ N/A – I have never experienced or witnessed a mistreatment

* 5. If you experienced or witnessed a mistreatment, did it interfere with the learning environment?

- ◯ Yes
- ◯ No
- ◯ N/A - I never experienced or witnessed a mistreatment

* 6. If you reported the mistreatment, to whom did you report it (check all that apply)?

- ☐ Dean
- ☐ Faculty member
- ☐ Department head
- ☐ Course coordinator
- ☐ Counselor
- ☐ Other medical school administrator
- ☐ N/A - I never experienced or witnessed a mistreatment
- ☐ Other (please specify)

* 7. If you did not report any incidents of mistreatment, what is the reason for NOT reporting them (check all that apply)?

- [ ] The incident did not seem important enough to report
- [ ] I resolved the issue myself
- [ ] I did not think anything could be done about it
- [ ] Fear of reprisal
- [ ] I did not know where to report it
- [ ] I reported all incidents
- [ ] There were no incidents to report
- [ ] Other (please specify)

Quality of Life

Rand short form survey intrument
Rand, 1776 Main Street, Santa Monica, California 90401-3208 (2016)

* 8. In general, would you say your health is:

- ◯ Excellent
- ◯ Very good
- ◯ Good
- ◯ Fair
- ◯ Poor

9. Compared to one year ago, how would you rate your health in general now?

- ◯ 1 - Much better now than one year ago
- ◯ 2 - Somewhat better now than one year ago
- ◯ 3 - About the same
- ◯ 4 - Somewhat worse now than one year ago
- ◯ 5 - Much worse now than one year ago

* 10. The following items are about activities you might do during a typical day. Does your health now limit you in these activities? If so, how much?

	Yes, limited a lot	Yes, limited a little	No, not limited at all
Vigorous activities, such as running, lifting heavy objects, participating in strenuous sports	◯	◯	◯
Moderate activities, such as moving a table, pushing a vacuum cleaner, bowling, or playing golf	◯	◯	◯
Lifting or carrying groceries	◯	◯	◯
Climbing several flights of stairs	◯	◯	◯
Climbing one flight of stairs	◯	◯	◯
Bending, kneeling, or stooping	◯	◯	◯
Walking more than a mile	◯	◯	◯
Walking several blocks	◯	◯	◯
Walking one block	◯	◯	◯
Bathing or dressing yourself	◯	◯	◯

* 11. During the past 4 weeks, have you had any of the following problems with your work or other regular daily activities as a result of your physical health?

	Yes	No
Cut down the amount of time you spent on work or other activities	◯	◯
Accomplished less than you would like	◯	◯
Were limited in the kind of work or other activities	◯	◯
Had difficulty performing the work or other activities (for example, it took extra effort)	◯	◯

* 12. During the past 4 weeks, have you had any of the following problems with your work or other regular daily activities as a result of any emotional problems (such as feeling depressed or anxious)?

	Yes	No
Cut down the amount of time you spent on work or other activities	◯	◯
Accomplished less than you would like	◯	◯
Didn't do work or other activities as carefully as usual	◯	◯

* 13. During the past 4 weeks, to what extent has your physical health or emotional problems interfered with your normal social activities with family, friends, neighbors, or groups?

◯ 1 - Not at all

◯ 2 - Slightly

◯ 3 - Moderately

◯ 4 - Quite a bit

◯ 5 - Extremely

* 14. How much bodily pain have you had during the past 4 weeks?

◯ 1 - None

◯ 2 - Very mild

◯ 3 - Mild

◯ 4 - Moderate

◯ 5 - Severe

◯ 6 - Very severe

15. During the past 4 weeks, how much did pain interfere with your normal work (including both work outside the home and housework)?

◯ 1 - Not at all

◯ 2 - A little bit

◯ 3 - Moderately

◯ 4 - Quite a bit

◯ 5 - Extremely

* 16. These questions are about how you feel and how things have been with you during the past 4 weeks. For each question, please give the one answer that comes closest to the way you have been feeling. How much of the time during the past 4 weeks..

	All of the time	Most of the time	A good bit of the time	Some of the time	A little of the time	None of the time
Did you feel full of pep?	◯	◯	◯	◯	◯	◯
Have you been a very nervous person?	◯	◯	◯	◯	◯	◯
Have you felt so down in the dumps that nothing could cheer you up?	◯	◯	◯	◯	◯	◯
Have you felt calm and peaceful?	◯	◯	◯	◯	◯	◯
Did you have a lot of energy?	◯	◯	◯	◯	◯	◯
Have you felt downhearted and blue?	◯	◯	◯	◯	◯	◯
Did you feel worn out?	◯	◯	◯	◯	◯	◯
Have you been a happy person?	◯	◯	◯	◯	◯	◯
Did you feel tired?	◯	◯	◯	◯	◯	◯

17. During the past 4 weeks, how much of the time has your physical health or emotional problems interfered with your social activities (like visiting with friends, relatives, etc.)?

◯ All of the time

◯ Most of the time

◯ Some of the time

◯ A little of the time

◯ None of the time

* 18. How TRUE or FALSE is each of the following statements for you.

	Definitely true	Mostly true	Don't know	Mostly false	Definitely false
I seem to get sick a little easier than other people	◯	◯	◯	◯	◯
I am as healthy as anybody I know	◯	◯	◯	◯	◯
I expect my health to get worse	◯	◯	◯	◯	◯
My health is excellent	◯	◯	◯	◯	◯

Mistreatment in Veterinary Education 2

Demographics

* 19. What is your age?

- 0-25 years of age
- 26-30 years of age
- 31-35 years of age
- 36-40 years of age
- >41 years of age

* 20. How confident to you feel in your ability to succeed in clinical veterinary medicine after graduation?

- Extremely confident
- Moderately confident
- Unsure
- Not very confident
- Not at all confident

* 21. What is your plan for the first year after graduation from veterinary school?

- Internship with the desire to specialize afterwards (residency)
- Internship followed by private practice
- Private practice
- Research of advanced degree (without any clinical work)
- Other

* 22. What is your gender?

- Male
- Female
- Other

* 23. In what year will you graduate veterinary school?

- ◯ 2017
- ◯ 2018
- ◯ 2019
